# The timing of vision in basketball three-point shots

**DOI:** 10.3389/fpsyg.2024.1458363

**Published:** 2024-10-30

**Authors:** Alessandro Piras

**Affiliations:** Department for Life Quality Studies, University of Bologna, Bologna, Italy

**Keywords:** motor control, attention, perception-action, expertise, eye tracking, microsaccades

## Abstract

The aim of the present study was to explore the relationship between gaze behaviour, motor responses and the direction of visual attention when different levels of basketball players were engaged in a basketball three-point shot. Twelve near-experts and 12 amateur basketball players, wearing an eye tracker and an inertial sensor, performed 20 shots on a basketball field, receiving the ball from a teammate, who then acted as the opponent. The trial sequence was subdivided into catching, aiming and ball flight phases. The analysis demonstrated that near-experts exhibited longer fixation durations and saccades of lower amplitude and peak velocity than amateurs. The gaze behaviour showed that all players utilized fixations during the last part of the catching phase, during most of the aiming phase, and during the final part of the ball flight phase. The greatest number of saccades was exhibited between the aiming and the ball flight phases, when the ball was released by the players. Saccades were oriented toward the teammate during the catching phase. Instead, during the aiming and ball flight phases, saccade orientations were not polarized toward a specific visual cue. In conclusion, vision plays a critical role in every aspect of the three-point shot in basketball, from catching the ball, to aiming preparation, and shot execution. It is a key factor in decision-making, spatial awareness, and overall performance in team sports.

## Introduction

Basketball is a dynamic and invasion sport, with quick movements and rapid decisions, influenced by visual skills, that involves the vestibular and somatosensory systems in focalizing the target. One of the fundamental skills of this team’s sport is the three-point shot, taken from beyond the three-point arc, which is a semicircle located 6.75 meters away from the basket in the FIBA rules. Teams often rely on proficient shooters to score points efficiently from long range. This has revolutionized the sport and contributed to the evolution of offensive schemes, with teams placing emphasis on perimeter shooting and spacing to maximize scoring opportunities (Lidor et al., 2022). In recent seasons, the three-point shot increased in popularity, suggesting that teams were increasingly pursuing offensive strategies aimed at three-point field goals with respect to the two-point shot (Romanowich et al., 2007; Gou and Zhang, 2022). This type of shot adds an extra strategic dimension to the game, as it rewards players who have developed long-range shooting accuracy and can extend defences, creating more space for their teammates to operate closer to the basket (Okazaki et al., 2015; Lidor et al., 2022).

Specific skills that impact shooting performance have been identified, such as anthropometric parameters of the player, shooting technique (height and velocity of ball release, angle of ball flight trajectory, distance from the basket), postural stability and fatigue (Okazaki et al., 2015). Nevertheless, to all these variables, another one that contributes to a better understanding of basketball shooting accuracy is the visual attention (Sirnik et al., 2022). The role of visual attention in the three-point shot in basketball is fundamental and multifaceted, impacting both the shooter and the defenders. Before attempting a three-point shot, a player needs to accurately locate the position of the hoop. Vision helps in focusing on the target and aligning the shot accordingly, playing a crucial role in assessing the distance from the hoop and deciding whether to take the shot, pass the ball or drive to the basket. Peripheral vision helps in making the right decision and recognizing teammates for possible passing, opposing, and positioning (Ryu et al., 2015; Ryu et al., 2013). During the execution of the shot, the shooter relies on visual feedback to ensure proper shooting form. This includes monitoring the trajectory of the ball, the alignment of the shooting arm, and the point of release. Both shooters and defenders need to recognize when a three-point shot opportunity arises. This requires rapid visual processing to assess the situation and react accordingly. In summary, vision plays a critical role in every aspect of the three-point shot in basketball, from catching the ball to shot preparation and execution. It is a key factor in decision-making, spatial awareness, and overall performance on the court (Vickers et al., 2019; Klostermann et al., 2018).

Most of the gaze behaviour studies in sports agree that experts look longer at relevant areas than their lower-level counterparts (for a review, see Mann et al., 2007; Zhang et al., 2022). Fewer fixations of longer durations are necessary to allow detailed parameterization for the required shooting movements. This is in accordance with other authors who deemed this period of fixation to be essential for programming the movement direction, force, and velocity, as well as limb coordination and timing (Timmis et al., 2023). Regardless of the type of eye movements, much attention has been dedicated to fixations, investigated as quiet eye, defined as a fixation or tracking gaze that is located on a specific object or location in the environment (for more information, see Vickers, 2021). Nevertheless, it has been found that our eyes are never stationary, even during fixations, because a kind of micro-movements, called microsaccades, continuously upset the gaze position (Martinez-Conde et al., 2013; Gu et al., 2024). These movements occur when athletes attempt to maintain steady fixation on a single point during a sports sequence (Piras et al., 2015). More recently, the interest in the role of microsaccades and other small saccades during fixation has increased, especially their role during action-perception tasks and the links with visuospatial attention (for a review, see Piras and Raffi, 2023).

During a basketball three-point shot, Vickers et al. (2019) demonstrated that experts tended to spend more time fixating on the centre of the hoop during arm flexion rather than arm extension or during ball release. Moreover, during arm flexion, the participants fixated on the hoop for the first time for a long duration, and this first fixation was initiated during the latter part of the arm preparation phase. As suggested by the authors, during the preparation phase, participants could have utilized the peripheral vision to catch the critical information (e.g., the position of the hoop). This reinforces the visual attention shift, in which central vision is used during the early part of fixations to identify cues, whereas peripheral information is used later in the fixation to select a target for the next saccade (Ryu et al., 2015). Therefore, we can hypothesize that experts, during the phase in which they receive the ball from the teammate, fixate on the player or on the ball, then shift their attention, with microsaccades or small saccades, toward the centre of the hoop just before the releasing of the ball. Bearing in mind the relationship between gaze behaviour, motor responses and the direction of visual attention, the current research investigated the role of gaze behaviour when different levels of basketball players were engaged in basketball three-point shots.

## Methods

### Participants

Twelve male near-experts and 12 male amateur basketball players volunteered to participate. Near-experts, with a mean age of 22 years old, played at the Serie B level (Italy championship); amateurs, with a mean age of 21 years old, played at the Serie D level (Italy championship) (see Table 1). Based on the sample size of the other studies (Piras et al., 2024; Vickers et al., 2019; de Oliveira et al., 2007; de Oliveira et al., 2006) and an effect size f of 0.35, G*Power (version 3.1.9.2) predicted that a total sample size of 24 would give appropriate power (1 − *β* error probability 0.90) to detect a significant difference at alpha level of 0.05. All players had normal or corrected-to-normal vision. After receiving oral and written information concerning the study protocol, all players signed the informed consent to participate in the study. The study was approved by the Bioethics Committee of our University.

**Table 1:** Athletes characteristics.

	Near-experts (12)	Amateurs (12)	*p*-value	Cohen’s *d*
Age (years)	21.58 ± 4.12	20.50 ± 1.31	0.401	0.35
Weight (kg)	88.50 ± 9.73	78.17 ± 7.07	0.005*	1.27
Height (cm)	192.42 ± 7.12	183.33 ± 7.16	0.007*	1.22
Body Mass Index (kg/m^2^)	23.83 ± 1.12	23.23 ± 1.21	0.221	0.51
Practice in basketball (years)	14.25 ± 5.15	13.92 ± 2.35	0.841	0.08
Training sessions (number/week)	7.25 ± 1.91	3.83 ± 0.72	0.000*	1.44
Training sessions (hours/week)	14.42 ± 3.12	7.75 ± 1.36	0.000*	2.40
Expertise level (2021–22)	B	D	\	\
Foot laterality	Right	Right	\	\
Hand laterality	Right	Right	\	\

*Significant different at *p* < 0.05.

### Apparatus

EyeLink II (SR Research), the video-based eye-tracking system, were used to record eye movements binocularly. The device consists of two miniature cameras mounted on a leather-padded headband. Pupil tracking is performed at 500 samples/s, with a gaze resolution of <0.005° and noise limited to <0.01°. The eye tracker was calibrated at the beginning of the experiment and after every 10 shots. Then, data validation and drift correction were performed by applying a corrective offset to the raw eye position data. Calibration and validation of the system were repeated every time a possible measurement error occurred due to participant movement. The accuracy of eye position was checked after every shot, and if necessary, a drift correction was performed. Practice, calibration, validation and data collection took about 20–30 min per participant.

In order to collect the exact time participants made each movement phase, one inertial sensor (Cometa Systems, Italy) was placed on the dorsal face of the right hand. Inertial sensors were synchronized with the external camera of the EyeLink II system to have correspondence between eyes, ball, and body movement data.

### Gaze behaviour data

Gaze behaviour consisted of fixations, saccades and microsaccades. Fixations were defined as the time the eyes remained stationary (within 1° of visual angle) for a minimum of 100 ms (Piras et al., 2014) and identified with the software Eyelink Data Viewer, that allows displaying, filtering, and presenting the results. Microsaccades were defined as micro-movements with <1° of visual angle, with a peak velocity smaller than 100°/sec, and with the same peak velocity versus amplitude curve as large saccades (Zuber et al., 1968). All eye movements that exceed 1° of visual angle and with a peak velocity >100°/s were identified as saccades. Microsaccades and saccades were considered if they occurred simultaneously in both eyes during at least 3 data samples (6 ms), and identified using the algorithms of Engbert and Kliegl (2003). Saccades and microsaccades rate, amplitude, duration, and peak velocity were calculated for each participant during each shot. Data were excluded 200 ms before and after each blink as well as when the pupil was still partially occluded (Piras et al., 2021b).

### Procedure

After a warm-up, a pre-test was performed without the eye tracker, followed by fitting the eye tracker and taking 3–5 practice trials until comfortable. Shots were taken from behind the three-point arc, which is a semicircle located 6.75 meters away from the hoop, on a regular basketball court used in competition. Placed right to the basketball hoop, wearing the Eye tracker and the inertial sensor, participants made 20 three-point shots interspersed by 5 min of rest after 10 shots. During each shot, participants received the ball from the teammate, who then acted as the opponent. The opponent had to actively challenge the participant, using an outstretched hand that was visible in the participant’s visual field during the trials. Participants were instructed to step forward to receive the ball and shoot, using the high-style shooting technique (Oudejans et al., 2002), as quickly as possible from behind the three-point line. Athletes were instructed and encouraged by their coach to do their best in each shot as if they were in a real match league.

### Statistical analysis

The length of the three-point sequence used for analysis was initially selected. The sequence started when the ball left the hands of the passer and ended when the ball reached the hoop. The sequence was subdivided into three epochs: catching the ball, aiming the hoop, and the ball flight. The catching phase began with the first frame of the video showing the ball leaving the hands of the passer and ended with the frame prior to the ball first contacting the hands of the player. The aiming phase began with the first frame showing the ball first contacting the hands of the player and ended with the frame showing the ball leaving the hands of the player. Then, the ball flight phase was started, which ended with the frame showing the ball passed or did not throw the hoop.

Response accuracy, the percentage of trials in which player’s response was correct or incorrect, was determined. It was analysed with repeated measure ANOVA, in which expertise (near-experts, amateurs) was the between-subjects factor, and response accuracy (correct, incorrect) was the within-subjects factor.

The phases of the movement were analysed with repeated measure ANOVA, in which expertise (near-experts, amateurs) was the between-subjects factor, phases of the movement (catching, aiming, ball flying) and response accuracy (correct, incorrect) were the within-subjects factors.

Repeated measure ANOVA was performed to analyse fixation numbers and durations. Expertise (near-experts, amateurs) was the between-subjects factor, phases of the movement (catching, aiming, ball flying) and response accuracy (correct, incorrect) the within-subjects factors.

Repeated measure ANOVA was performed to analyse saccade and microsaccade rate, amplitude, duration, and peak velocity. Expertise (near-experts, amateurs) was the between-subjects factor, phases of the movement (catching, aiming, ball flying) and response accuracy (correct, incorrect) the within-subjects factors.

The two-dimensional distribution of all saccade and microsaccade orientations was calculated based on expertise (near-experts, amateurs) and phases of the movement (catching, aiming, ball flying). The Watson-Williams test for homogeneity of means (Oriana^®^ 4.0) was performed. The null hypothesis was that the orientations of saccades and microsaccades between expertise and phases of movement have similar continuous distribution at the 5% significance level.

Finally, we also calculated the time course of the saccade rates (N°/s) for the entire duration of the trial and subdivided into phases: catching, aiming, and ball flight. Rates were computed for each basketball player and subdivided into near-experts and amateurs using a moving time window of 200 ms. The shaded area around each curve represents the standard error of the mean.

All statistical analysis was done with SPSS, version 22.0 (Chicago, IL, USA). Effect sizes were calculated as the mean difference standardized by the between-subject standard deviation and interpreted according to the following thresholds: trivial, <0.20; small, ≥0.20 < 0.50; moderate, ≥0.50 < 0.80; large, ≥0.80 (Cohen, 1988). Partial eta squared ( $\eta_{p}^{2}$ ) was used during multiple comparisons. Statistical significance was set at *p* < 0.05. *Post hoc* testing was corrected using the Bonferroni procedure.

## Results

All players made more incorrect (mean 11.75 ± 2.4; 59%) than correct (mean 8.25 ± 2.4; 41%) three-point shot (*F*_1,22_ = 12.6; *p* = 0.002; $\eta_{p}^{2}$ = 0.36). No significant differences were observed for expertise (*p* = 0.41; $\eta_{p}^{2}$ = 0.03).

**Figure 1:**
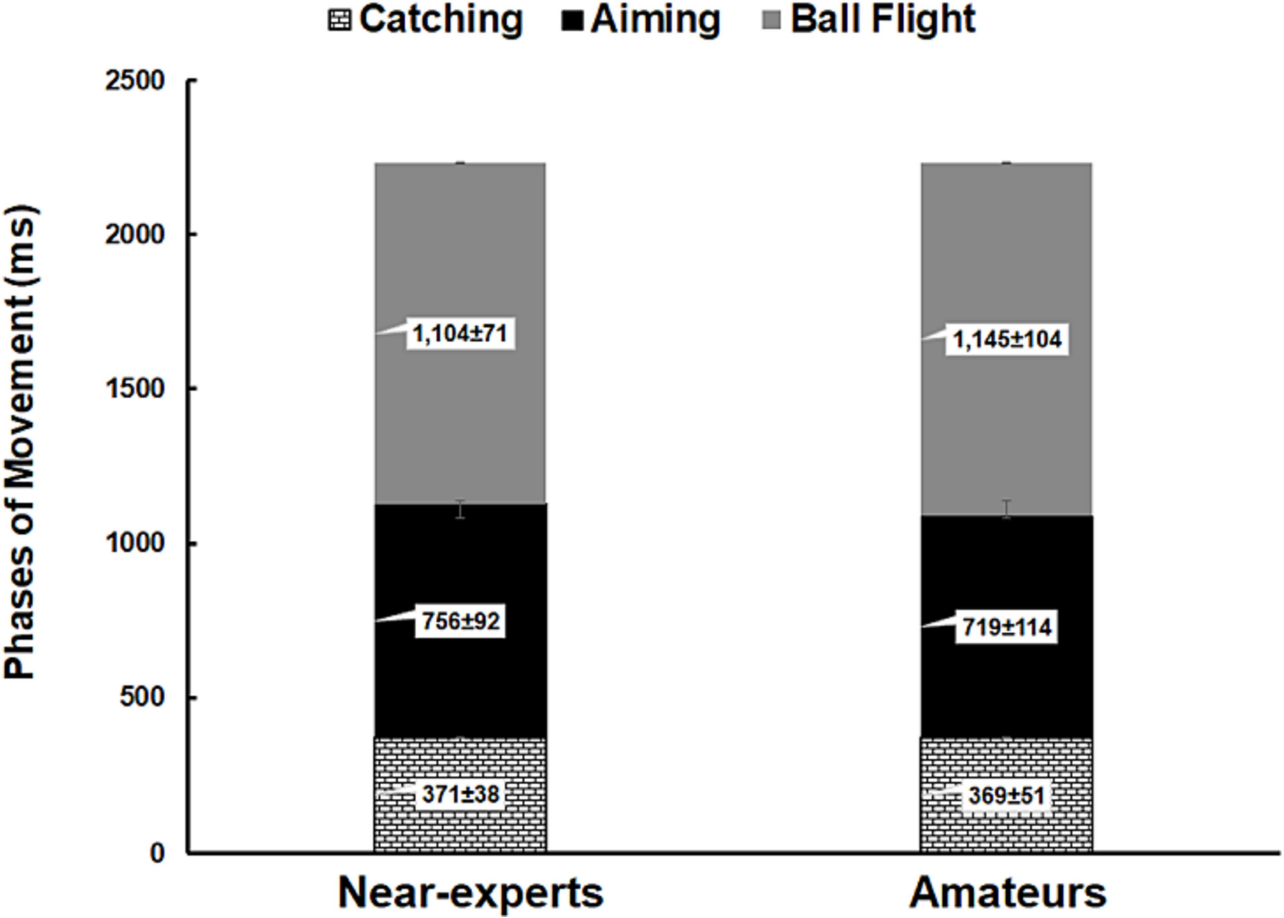
Histograms represent the phases of movement (mean ± SD) subdivided into catching (white squares), aiming (black) and ball flight (grey) in both near-experts and amateur basketball players.

Analysis of the phases of movement showed significant differences between phases (*F*_2,44_ = 492.2; *p* < 0.001; $\eta_{p}^{2}$ = 0.96), in which all players spent more time on ball flight phases and less time during catching (see Figure 1). No significant differences were detected for expertise or response accuracy (*p* > 0.05).

**Figure 2:**
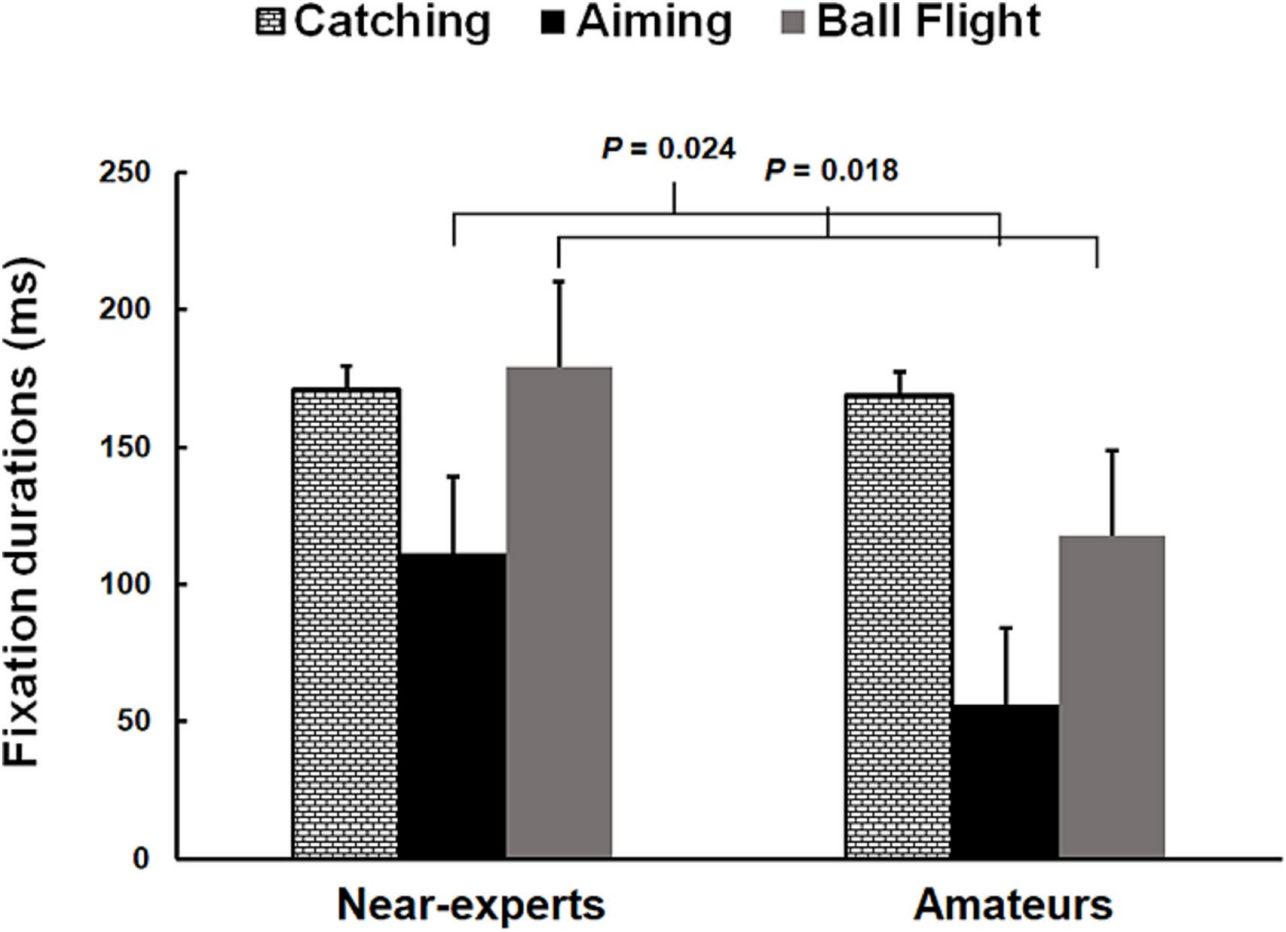
Histograms represent values (mean ± SD) of fixation durations (msec) in catching (white squares), aiming (black) and ball flight (grey) between near-experts and amateur basketball players. The horizontal bands represent multiple comparisons at *p* < 0.05.

Fixation durations showed significant differences for expertise (*F*_1,22_ = 5.8; *p* = 0.024; $\eta_{p}^{2}$ = 0.21), between phases (*F*_2,44_ = 20.5; *p* < 0.001; $\eta_{p}^{2}$ = 0.48) and also for the interaction between expertise and phases (*F*_1,22_ = 3.7; *p* = 0.042; $\eta_{p}^{2}$ = 0.16). Post-hoc analysis showed that near-experts made longer fixation durations than amateurs, and it was more evident during aiming and ball flight (see Figure 2). No significant differences were observed for response accuracy (*p* > 0.05).

The number of fixations exhibited significant differences for phases, with the highest value displayed during ball flight and the lowest during aiming. No significant differences were observed for expertise, nor for response accuracy (*p* > 0.05).

The Engbert and Kliegl (2003) algorithm to identify saccades and microsaccades reported only saccades >1 degree of visual angle and faster than 100 degrees/sec. Indeed, we did not find microsaccades as they are defined by the literature (Martinez-Conde et al., 2009).

Analysis on saccade rates showed significant differences for expertise (*F*_1,22_ = 4.9; *p* = 0.044; $\eta_{p}^{2}$ = 0.11), between phases (*F*_2,44_ = 29.1; *p* < 0.001; $\eta_{p}^{2}$ = 0.57), and for their interaction (*F*_2,44_ = 3.2; *p* = 0.046; $\eta_{p}^{2}$ = 0.10). Near-experts made fewer saccades than amateurs, and this was particularly evident during catching the ball. All players exhibited the greatest saccade rates during ball flight and the lowest during aiming. No significant differences were observed for response accuracy (*p* > 0.05).

**Figure 3:**
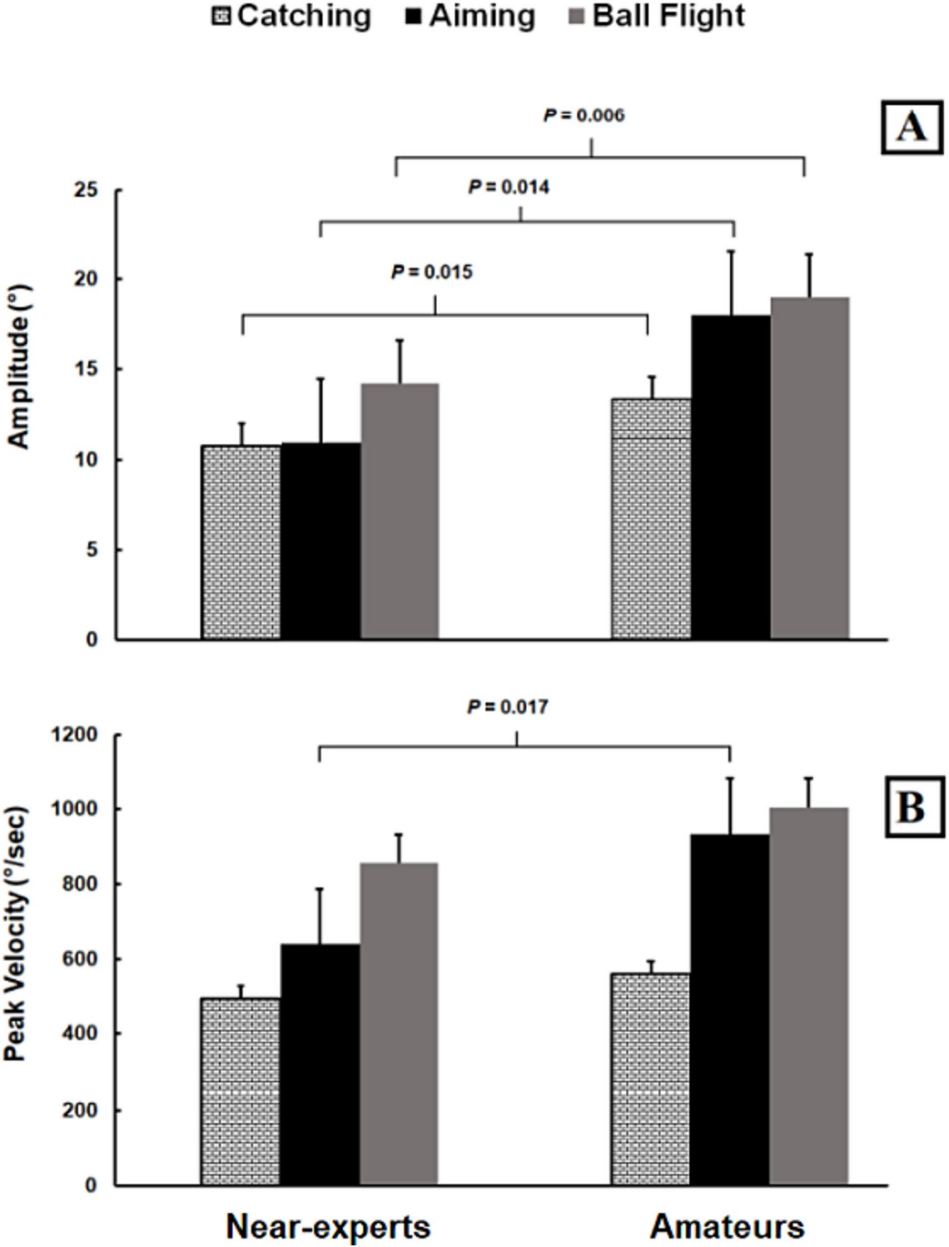
Histograms represent values (mean ± SD) of saccade amplitude **(A)** and peak velocity **(B)** in catching (white squares), aiming (black) and ball flight (grey) between near-experts and amateur basketball players. The horizontal bands represent multiple comparisons at *p* < 0.05.

Saccade amplitudes showed significant differences for expertise (*F*_1,22_ = 20.4; *p* < 0.001; $\eta_{p}^{2}$ = 0.48) and between phases (*F*_2,44_ = 6.5; *p* = 0.003; $\eta_{p}^{2}$ = 0.24), but not for their interaction (*p* > 0.05). Near-experts made saccades of lower amplitude in comparison to amateurs, and this happened during all phases (see Figure 3A). All players exhibited the greatest saccade amplitude during ball flight and the lowest during catching. No significant differences were observed for response accuracy (*p* > 0.05).

Saccade peak velocities showed significant differences for expertise (*F*_1,22_ = 5.3; *p* = 0.031; $\eta_{p}^{2}$ = 0.19) and between phases (*F*_2,44_ = 20.8; *p* < 0.001; $\eta_{p}^{2}$ = 0.48), but not for their interaction (*p* > 0.05). Near-experts made saccades of lower peak velocity in comparison to amateurs, and this was more evident during the aiming phase (see Figure 3B). All players exhibited the greatest peak velocity during ball flight and the lowest during catching. No significant differences were observed for response accuracy (*p* > 0.05). No significant differences were observed for saccade durations (*p* > 0.05).

**Figure 4:**
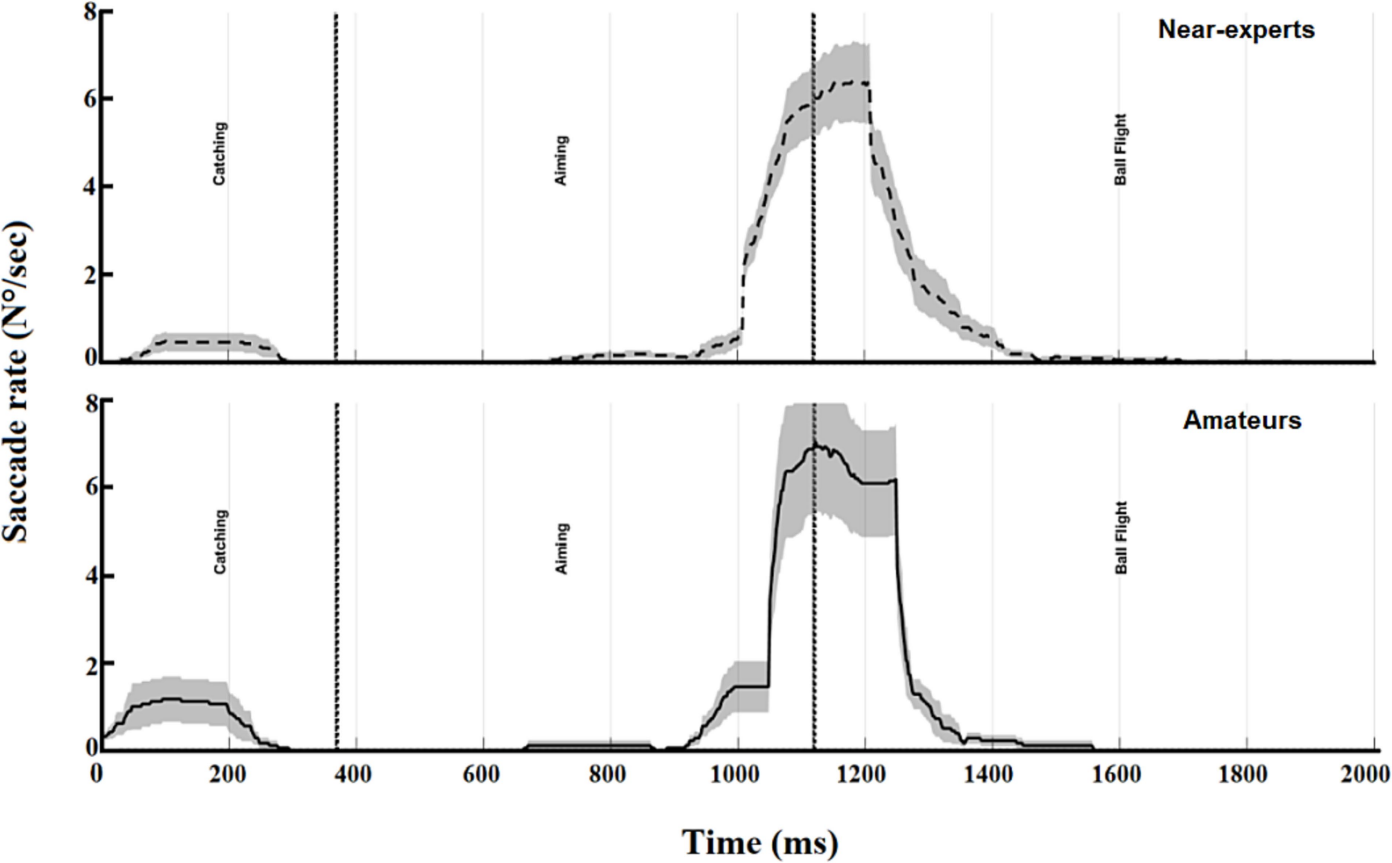
The time course of the saccades rate (N°/sec) was calculated for the entire duration of the trial and subdivided into phases: catching, aiming, and ball flight. Rates were computed for each basketball player and subdivided into near-experts (upper panel, dashed line) and amateurs (lower panel, solid line) using a moving time window of 200 ms. The shaded area around each curve represents the standard error of the mean.

The time course of saccade onset showed a different trend during phases (Figure 4). The greatest values related to the onset of the saccades were observed during the end of the aiming phase and the start of the ball flight phase, at the moment in which the ball was leaving the players’ hands toward the basket. The absence of the saccade onset between the catching and aiming phases highlighted the need for the players to maintain stable fixation on the target just before to start the shooting movement.

**Figure 5:**
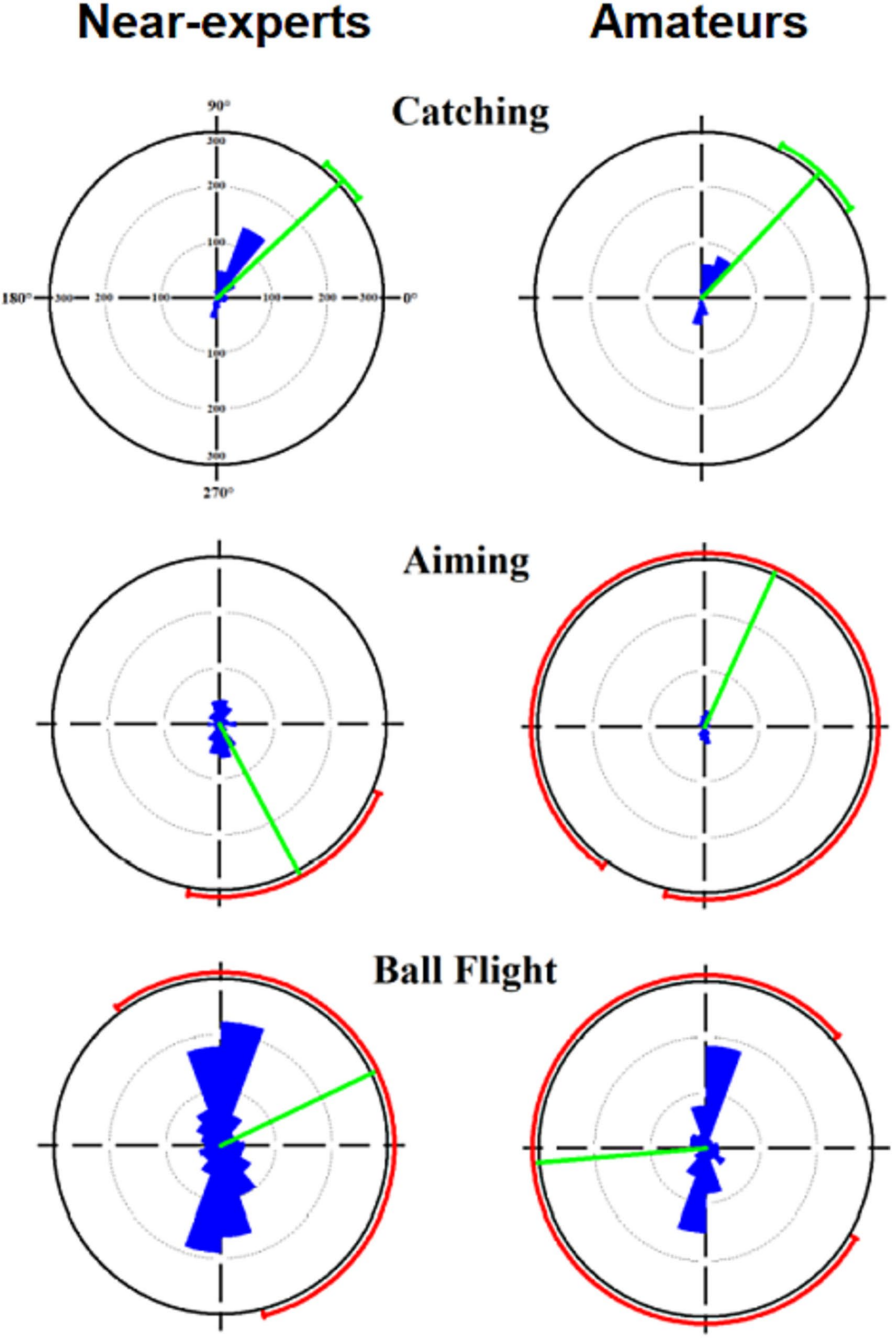
Panels represent the mean vector direction of saccades during catching, aiming and ball flight between near-experts (left panels) and amateur (right panels) basketball players. Each angular sector is 20° in width. Green radial thick is the mean vector, and the red ring outside the polar plot is the standard deviation.

Saccades orientation showed no significant differences between near-experts and amateurs across movement phases (*p* < 0.05). As shown in Figure 5, while catching the ball, saccades showed a main vector directed to the upper-right of the players’ visual field. Meanwhile, the direction of the main vector was inaccurate during the other two phases, showing a greater standard deviation (the ring dimension outside the polar plot, Figure 5), which means that the saccade orientations were not polarized toward a specific direction.

## Discussion

The aim of the current research was to investigate the role of gaze behaviour when different levels of basketball players were engaged in basketball three-point shots. This is the first study that has tried to record saccades and microsaccade characteristics during a far-aiming task in a constrained approach, in which athletes were engaged in shooting action against a defender who tried to stop the attack. We did not find microsaccades as they are defined by the literature, which are eye movements inside 1° of visual angle and with a peak velocity lower than 100°/sec. Our data revealed that, in the absence of a foveal target to fixate on and due to the fast sporting sequence, our athletes did not make microsaccades, opting for eye movements of greater amplitude and peak velocity, supporting the suggestion that microsaccades necessitate the presence of a “*target to anchor to*” (Otero-Millan et al., 2008). The absence of a fixation target, or when it is larger than the fovea area, has an effect on the microsaccades, decreasing the rates and increasing the amplitudes (McCamy et al., 2013; Otero-Millan et al., 2019). This is possible because the change of the fixation area through phases forced athletes to move their eyes at great retinal eccentricities. Probably, during a far-aiming task, the athletes’ visual search strategy shows more fixations of shorter duration with respect to near or interceptive tasks. This extensive number of perceptual information sources, located disparately across a large field area, constrain the athletes to employ more frequent fixations than in the micro-state contexts (Williams and Davids, 1998). More recently, in soccer goalkeepers, Piras et al. (2021a) found that during penalties kicked from 11 meters, goalkeepers used a visual search strategy with more fixations and consequently greater saccade rates in comparison to penalties kicked from 6 meters, where they exhibited fewer fixations and higher microsaccade rates. In this article, we can only speculate it, even because the soccer goalkeepers performed an interceptive timing task, where the gaze fixates and/or tracks an object moving toward them that must be controlled (receiving the ball). In our study, gaze strategy has been investigated in an aiming task (throwing a ball), in which the gaze fixates on a critical target location(s) before an object is aimed away from the body. In the previous studies that have investigated the role of microsaccades in sports, a fixation target was always present; the fixation point in table tennis (Piras et al., 2015; Piras et al., 2019) and the ball in soccer goalkeepers (Piras et al., 2021b; Piras et al., 2021a). This investigation differs from the others that have studied the role of microsaccades in a sports context. This experiment followed an ecological approach, with a dynamic sequence, under constraints imposed by the opponent, in a far-aiming task.

Another potential explanation for the absence of microsaccades is that movement intention suppresses their occurrence (Betta and Turatto, 2006), especially in a dynamic, information-rich environment where microsaccades may interfere with critical perception. Maybe we will need more cutting-edge instruments to investigate this tiny eye movement, or maybe they are suppressed when the movements of the entire body in a far-aiming task are rapid, ballistic, and involved in a constrained approach. We need to look deeply inside the role of microsaccades in some contexts, such as dynamic interceptive tasks, where the concept of microsaccades might indicate increased focus, heightened preparatory states, or their capacity to achieve a state of readiness before upcoming tasks. The implication is that eye movement registration techniques are slightly limited to indicating the locus of fixation and not necessarily the locus of attention in dynamic team sports situations (Piras, 2025). The role of sports science research is to clarify the relationship between the location of perceptual sources of information, the specific task constraints, and the type of gaze behaviour engaged by the athletes. More work is needed to investigate the further impact of using microsaccades to develop the ability to locate and identify visual information in the environment for the selection and execution of actions.

In dynamic far-aiming tasks, like shooting a soccer ball or during basketball jump shooting, there is often no time for long fixations and, therefore, no time to process a motor program (de Oliveira et al., 2006). In this dynamic sequence, the timing for catching and detecting visual information is critical and limited by the constraints, so the visual search strategy should be used online. Adopting the high-style shooting, the ball is first positioned overhead, followed by the elbow extension until the ball is released. An advantage of the high style is that the shooter can look at the basket from underneath the ball when it is held overhead (Oudejans et al., 2002), allowing online visual control of the final shooting movements between the aiming and the ball flight phases. As a result, players need to pick up the necessary information at precise time intervals at which the information is visually available and can be used to control the action. In the present study, basketball jump shooters prefer to pick up optical information about the basket, maintaining a stable fixation between the end of the catching phase and during most of the aiming phase until the ball is released. Then, just before the ball leaves the athletes’ hands, the saccade rates increase exponentially, reaching the highest values (see Figure 4). This could be related to the next phase present in a real match, when the player, after the shot, is ready for the rebound. This happens when the ball hits the rim or the backboard, and then the players fight for the best position to snatch the rebound. To get the rebounding ball, the player is required both to perceive temporal and spatial information through a complex visual field and to react to an opponent player immediately. As illustrated in Figure 5, saccade directions were not informative, except for the catching phase in which players directed their attention toward the player who passed the ball. During aiming and ball flight, the saccade orientations were not uniform, meaning that they did not have a particular direction. Assuming that with the high shooting style, visual information is available until ball release, we expected that gaze fixation could be used for the visual control of the jump shot. The three-point shot is very difficult and fast; from the moment the ball is received until it leaves the hands, elite players release the ball in 600–800 ms, increasing the difficulty for the opponent to intercept it (Vickers et al., 2019). Different studies have investigated the role of vision during jump shots in basketball, and they have ended with different conclusions (Oudejans et al., 2002; de Oliveira et al., 2008; Vickers et al., 2019; Vickers, 1996). One of the first was that of Oudejans et al. (2002), who compared early vs. late vision information when athletes adopted the high shooting style. The authors found that performance with early vision was severely impaired with respect to late visual information. They concluded that when players used a high-style, they raised the ball above their heads and acquired late visual information from the target prior to and during ball release. The final shooting movement is controlled by continuous detection and the use of visual information until the ball is released. A few years later, other authors (de Oliveira et al., 2008), comparing high vs. low shooting style, supported the view that basketball shooting is mainly controlled online by vision, meaning that visual information is picked up and used during movement, rejecting the suppression of the vision during the final shooting phase (Vickers, 1996). More recently, Vickers et al. (2019) have investigated the timing of fixations (early, late), the location of fixations (hoop centre, non-centre) and the effect of the defender during three-point basketball shooting. They found that during the defended condition, the trial duration and the response accuracy were lower than during the undefended condition. Moreover, Vickers et al. (2019) found a low number of fixations during ball release, showing that the three-point shot is taken under such extreme time and defensive pressure that sustaining a fixation until the ball is released is very difficult.

The results of the present study are in accordance with that of Vickers et al. (2019), with a trial duration of about 1,200 ms (without considering the ball flight) and a response accuracy of about 41%. Furthermore, as Figure 4 suggested, the saccade rate showed the highest value during ball release. The three-point shot in basketball is an open skill performed in a movement patterns that is variable and unpredictable and requires athletes to adapt their body movements in response to the dynamic characteristics of the environment. Being a dynamic and constrained task, athletes are subjected to pressure, which can negatively compromise their expectations. More specifically, pressure changes the athletes’ attentional mechanisms and memory processes that support performance (Lidor et al., 2022). This phenomenon alters the visual search strategy, observed moving from the first phase in which performers receive the ball without pressure, with a stable fixation and gaze behaviour focused in a particular location, to ball release, in which the opponent pressure increases the instability of the athletes’ eyes, with higher saccade rates, amplitudes and peak velocities.

Within the design of the current study, we acknowledge the lack of significant differences in accuracy. This is the main limitation of the current study, related to one of the common problems presented in sports performance: the difficulty of finding players at a very high level (Vickers et al., 2019; Piras et al., 2024). For example, in the National Basketball Association, the number of players that have exhibited a score > 40% in the previous season (2023–24) is only 36 in front of about 560 athletes. The 3-point-field percentage of Stephan Curry was 48.1%.^1^

In conclusion, the results of the present study showed that near-experts exhibited longer fixation durations in both aiming and ball flight phases, with saccades of lower amplitude and peak velocities than amateurs. This result may suggest that these experts have developed an ability to control their eye movements, likely unconsciously, to optimize the visual selection process. Additionally, they seem able to tolerate any potential distractions caused by movement, minimizing its impact on their performance. The time course of saccade rates showed that athletes utilize fixations during the last part of the catching phase and during most of the aiming phase, making the greatest number of saccades when the ball release started. Saccade orientations were not informative, maybe because the change of the fixation areas through phases, forced athletes to move their eyes at great retinal eccentricities. This study can be considered a completion of previous studies regarding the timing of vision during a basketball shooting. In the present results, fixations occur early in the shooting action (Vickers et al., 2019; Klostermann et al., 2018), and following an ecological setting, it supported the importance of acquiring late visual information from the target prior and during ball release (de Oliveira et al., 2006; de Oliveira et al., 2008; de Oliveira et al., 2007).

## Data Availability

The datasets presented in this article are not readily available because as the nature of this research is within a high-performance environment, athletes of this study did not agree for their data to be shared publicly, so supporting data are not available. Requests to access the datasets should be directed to AP, alessandro.piras3@unibo.it.

## Footnotes

^1^https://www.nba.com/stats/player/201939
